# Introduction of the Probang into the Larynx

**Published:** 1858-01

**Authors:** F. D. Leute

**Affiliations:** Cold Spring


ART. VIII.—*Introduction of the Probang into the Larynx and Medicated Fluids into the Trachea.* By F. D. LEUTE, M.D., of Cold Spring.

THE question as to the possibility of introducing medicated solutions into the larynx and trachea for the relief of diseases of these organs seems to be still open. At first it was denied that the sponge could be passed into the larynx; subsequently this was generally admitted to be practicable. The impression is still, however, almost universal that the sponge cannot be passed through the chordæ vocales into the trachea. But I was very much surprised to see, in a recent periodical, a quotation from one of Trousseau's late lectures, in which he absolutely denies the possibility of even passing a sponge into the upper opening of the larynx. He says he cannot do it, and that no other man ever did it,—to such a pitch will prejudice carry one. About the year 1848, as nearly as I can recollect, I had a case under my care, in the New York Hospital, which completely settles this latter question, and, to a certain extent, the former also.

A patient in ward 9 of the first Surgical Division (according to the old arrangement of the wards) was recovering from a self-inflicted wound of the throat, which had opened the trachea, only a small fistulous opening being left, when he was attacked with aphonia, for the relief of which I was directed to apply a strong solution of nitrate of silver by means of the sponge and probang. I applied this several times, with a view of passing the solution into the larynx, and finally with complete relief, if I remember rightly, to the aphonia. But what I do remember clearly is that, on two occasions at least, after performing the operation, the skin around the fistulous opening in the neck was *stained distinctly with the nitrate of silver* which had mingled with the usual mucous discharge from the interior of the trachea.

This case then establishes the fact that it is practicable to introduce the sponge into the larynx, and to introduce medicated solutions (I do not say the sponge) into the trachea.

